# In-silico predictive mutagenicity model generation using supervised learning approaches

**DOI:** 10.1186/1758-2946-4-10

**Published:** 2012-05-15

**Authors:** Abhik Seal, Anurag Passi, UC Abdul Jaleel, Open Source Drug Discovery Consortium, David J Wild

**Affiliations:** 1 Indiana University Bloomington School of Informatics and Computing, Bloomington, USA; 2 Open Source Drug Discovery, Council of Scientific and Industrial Research, New Delhi, India; 3 Department of Cheminformatics, Malabar Christian College, Kerala, India

**Keywords:** Molecular descriptors, Machine learning, Mutagenicity, Random forest, Screening, Toxicophores

## Abstract

**Background:**

Experimental screening of chemical compounds for biological activity is a time consuming and expensive practice. *In silico* predictive models permit inexpensive, rapid “virtual screening” to prioritize selection of compounds for experimental testing. Both experimental and *in silico* screening can be used to test compounds for desirable or undesirable properties. Prior work on prediction of mutagenicity has primarily involved identification of toxicophores rather than whole-molecule predictive models. In this work, we examined a range of *in silico* predictive classification models for prediction of mutagenic properties of compounds, including methods such as J48 and SMO which have not previously been widely applied in cheminformatics.

**Results:**

The Bursi mutagenicity data set containing 4337 compounds (Set 1) and a Benchmark data set of 6512 compounds (Set 2) were taken as input data set in this work. A third data set (Set 3) was prepared by joining up the previous two sets. Classification algorithms including Naïve Bayes, Random Forest, J48 and SMO with 10 fold cross-validation and default parameters were used for model generation on these data sets. Models built using the combined performed better than those developed from the Benchmark data set. Significantly, Random Forest outperformed other classifiers for all the data sets, especially for Set 3 with 89.27% accuracy, 89% precision and ROC of 95.3%. To validate the developed models two external data sets, AID1189 and AID1194, with mutagenicity data were tested showing 62% accuracy with 67% precision and 65% ROC area and 91% accuracy, 91% precision with 96.3% ROC area respectively. A Random Forest model was used on approved drugs from DrugBank and metabolites from the Zinc Database with True Positives rate almost 85% showing the robustness of the model.

**Conclusion:**

We have created a new mutagenicity benchmark data set with around 8,000 compounds. Our work shows that highly accurate predictive mutagenicity models can be built using machine learning methods based on chemical descriptors and trained using this set, and these models provide a complement to toxicophores based methods. Further, our work supports other recent literature in showing that Random Forest models generally outperform other comparable machine learning methods for this kind of application.

## Background

In the past two decades high throughput screening (HTS) has provided a large amount of experimental data on compound biological activities. Data mining and machine learning methods provide an *in silico* counterpart building predictive models based on chemical structure features and other properties, and training sets of known bioactivities. Despite these capabilities quantitative methods do not tend to model the biochemical and physiological process well. Recent developments in machine learning have focused on the exploration of large data sets with non–congeneric molecules. The applicability of Quantitative Structure Activity Relationship (QSAR) studies to predict toxicity is very limited. The rationale behind the use of machine learning is to discover patterns and signatures in data sets from high throughput *in-vitro* assays. Nonetheless, the development of *in-silico* models as alternative approaches to mutagenicity assessment of chemicals without animal testing is constantly increasing and has attracted researchers in the field of Quantitative Biological Activity Relationship (QBAR) [1] and even toxicology.

Mutagenicity is the ability of a substance to cause genotoxicity. Experimentally, mutagenicity is assessed by Ames test performed on *Salmonella typhimurium* bacterial strains where each bacterial strain is sensitive to specific chemical mutagen [2]. It has been found that the predictive power of positive Ames test for rodent carcinogenicity is high, ranging from 77% to 90% [3]. Kazius et al. [4] assembled a data set of 4337 compounds and derived 29 toxicophores with an error rate of 18% in training set and 15% in a validation test set. Helma et al. [5] reported MOLFEA algorithm for generation of descriptors based on molecular fragments for non-congeneric compounds and compared various machine learning algorithms with its data set of 684 compounds derived from Carcinogenic Potency Database (CPDB: http://potency.berkeley.edu/). The data set gave an accuracy of 78% with 10 folds of cross validation. Hansen et al. [6] reported a unique new public Ames Mutagenicity data set with 6500 compounds and compared results with commercial and non-commercial tools. Zhang and Sousa [7] also reported the use of MOLMAP descriptors for bond properties which were used for training of Random Forest classifier. Error percentages, as low as 15% - 16% were achieved with an external validation set of 472 compounds against a training set of 4083 structures. Up to 91% sensitivity and 93% specificity were obtained from the test sets. Feng et al. [8] used four data sets NCI, Mut, Yeast and Tox and generated four different types of descriptors. Using statistical methods, models were built to link chemical descriptors to the biological activity. King et al. [9] reported different methods for establishing structure activity relationships (SARs). They represented chemical structures by atoms and bond connectivities in combination with inductive logic programming algorithm Progol. They tested 230 compounds which were divided in two sets of 188 compounds and 42 compounds. For 42 compounds Progol formed a SAR better than linear regression and back propagation. Judson et al. [10] used different classifiers to predict the accuracy of the model of complex chemical toxicology data sets. Neural networks and Support Vector Machines (SVM) were at the top of the list of classifiers, predicting with 96% and 99% specificity, respectively. They also mentioned that irrelevant features decreased the accuracy rate, with linear discriminant analysis suffering the maximum degradation. Ferarri and Gini [11] proposed the idea of a trained QSAR classifier supervised by a SAR layer that incorporates coded human knowledge. The model is implemented in the CAESAR project (http://www.caesar-project.eu) [12] where initially a classifier is trained on more than four thousand molecules based on Bursi data set by using molecular descriptors, then in the next step the relative knowledge to complement its practice is extracted from a collection of well-known structural alerts. Votano et al. [13] reported the application of three QSAR methods using artificial neural networks, *k*-nearest neighbors, and decision forest, to a data set of 3363 diverse compounds. They used molecular connectivity indices, electrotopological state indices, and binary indicators to obtain an accuracy of 82%.

Unlike many bioactivities, mutagenicity can be linked to very specific chemical structure fragments and functional groups, usually referred to as toxicophores, which interfere with DNA [14-16]. These include aromatic amines, hydroxyl amines, nitroso compounds, epoxides, thiols, nitrogen mustards, aziridines, aromatic azo’s, propiolactones, aliphatic halides, thiophenes, heteroatom derivatives, polycylic planar compounds, hydrazine, hydrazide and hydroxylamine. It has also been found that detoxifying structures such as the CF3, SO2NH, SO2OH and aryl sulphonyl derivates render mutagenic compounds non-mutagenic [17].

In this paper, firstly, we have applied four classification algorithms - Naïve Bayes, J48, Random Forest and Sequential Minimal Optimizer (SMO) - to model the mutagenicity data of compounds. In particular, we were interested in discovering whether such “whole molecule” algorithms are appropriate for mutagenicity prediction, or whether this is better done using simple alerts based on toxicophores. We were also interested in whether we would replicate previous work indicating that Random Forest is a better classifier than other Base and Ensemble classifiers [18]. We tested the model with validation sets (PubChem data sets AID1189 and AID1194, DrugBank [19] approved, and withdrawn drugs and Zinc metabolites data (zinc.docking.org/browse/subsets/special.php) [20] all of which indicate that the Random Forest model performs well.

## Methods

### Data sets

This work included 3 training data sets: Set 1 (Bursi mutagenicity data set) having a total of 4337 compounds, Set 2 (Benchmark data set) with 6512 compounds and Set 3 which was a combination of Set 1 and Set 2 containing 8208 compounds after removing the duplicate structures based on the canonical smiles of the Set 1 and Set 2 using Pipeline Pilot [21]. The data sets were divided into training (80%) and testing (20%). The datasets are given in the Additional file 1 and Additional file 2. Table 1 shows the distribution of compounds on the training and test sets of the three sets (Set 1, Set 2 and Set 3). For using the datasets in Weka we performed the remove useless feature option which removes the unnecessary variables from the data. We converted the data to the ARFF format for further classification. For set 1 “remove useless” operation in Weka, of the initial 179 descriptors, 151 descriptors which contained 24 weighted burden number descriptors, 8 properties descriptor and 120 pharmacophore fingerprints were obtained. For set2 and 3 the remove useless operation resulted in 154 descriptors (of the initial 179 descriptors) which contained 24 weighted burden number descriptors, 8 properties descriptors and 123 pharmacophore fingerprints.

**Table 1:** Distribution of different data sets and it compounds (mutagens and non-mutagens) in test and train sets

**Data sets**	***Training***	***Training***	***Test mutagen***	***Test non***	***Minority %***
	***Mutagen***	***Non mutagen***		***Mutagen***	
**Set 1**	1916	1554	485	382	55.38
**Set 2**	2803	2407	700	602	53.79
**Set 3**	3639	2871	910	788	55.40

For validation of the generated model, external test sets were used. External data sets, AID1189 and AID1194, were taken from EPA DSSTOX data set in the CPDB [22]. AID1189 contained 1477 compounds with 788 mutagens and 689 non-mutagens and AID1194 contained 832 compounds with 396 mutagens and 436 non-mutagens. The toxicity models were tested against the 1410 approved drugs and 66 withdrawn drugs from the DrugBank database and as well as with the 22080 metabolite data which were taken from the recently published ZINC Data sets. The metabolites may be toxic or non-toxic the idea here is to check whether the compounds formed after metabolism has some mutagenicity or not using our predictive models.

### Chemical descriptors

For each data set, descriptors were calculated by PowerMV [23]. PowerMV calculates a total of 6122 descriptors classified as 546 atom pair descriptors, 4662 Carhart descriptors, 735 fragment pair descriptors, 147 pharmacophore fingerprints, 24 Weighted Burden Number descriptor and 8 properties descriptors. Among those we used:

*Property descriptors* including XlogP (a measure of the propensity of a molecule to partition into water or oil), polar surface area (PSA), number of rotatable bonds, H-bond donors, H-bond acceptors, molecular weight, blood–brain indicator (0 indicating a compound does not pass the BBB, and 1 indicating that a compound passes the BBB) and bad group indicator (the molecule contains a chemically reactive or toxic group).

*Pharmacophore Fingerprint descriptors* based on bioisosteric principles. They are divided in to six classes totaling to 147 descriptors.

*Weighted Burden number descriptors*, a set of continuous descriptors and are also a variation of the Burden number [24]. One of the three properties, namely, electronegativity, Gasteiger partial charge or atomic lipophilicity and XLogP is placed on the diagonal of the Burden connectivity matrix. The off-diagonal elements are weighted by one of the following values: 2.5, 5.0, 7.5 or 10.0. Then the largest and the smallest eigenvalues are used as descriptors.

### Machine learning classifiers

Machine learning has been widely used in classifying molecules as active or inactive, mutagen or non-mutagen against a protein target [25]. In this work we used Weka [26] open source software which is a collection of different classifiers for data mining and machine learning. It is licensed under GNU GPL. It includes tools for data pre-processing, classification, regression, clustering, association rules, and visualization. Of the many data mining approaches that have been explored, four have evolved to largely dominate other classification methods at present. These are a) Bayesian methods [27] b) Support Vector Machines [28] c) Decision trees [29] and d) Random Forest [ 30,31].

### Workflow

**Figure 1:**
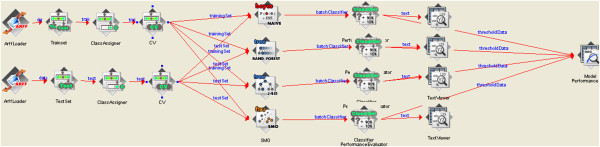
The diagram above represents the knowledge workflow model of Weka environment software.

The data sets were downloaded in SD File format. The PowerMV descriptor calculation tool was used to generate chemical descriptors. A total of 179 descriptors were generated for all the three data sets used. Bioassay data was appended as an outcome column to each of the data sets in the comma separated values (CSV) file format. The compounds were labeled mutagens and non-mutagens based on the respective bioassay data. After merging of Set1 and Set 2 compounds, the duplicate compounds are removed from the data and it resulted in 8292 compounds representing Set 3 data set. Useless descriptors were removed among the 179 descriptors which resulted in 155 descriptors for Set 2 and Set 3 and 152 descriptors for Set 1. Each data set was trained with 10 fold cross validation with default parameters for all the four classifiers mentioned earlier. The models generated were tested with remaining 20% test data and also validated using external data sets from PubChem AID1189 and AID1194, DrugBank drugs and Zinc metabolites data. Additional file 3 contains the csv formatted file of descriptors for external datasets. Using the knowledge flow provided by Weka, a workflow represented in Figure 1 was prepared which loads the data sets, applies the classifiers to generate the models which are tested using the test compounds.

## Results

The results are discussed for each of the data sets for which the models were developed using the four classifiers. The Random Forest was parameterized with 100 trees because we did not find much difference in the out of bag error rates for 500 trees (which was around less than 0.5%).

**Figure 2:**
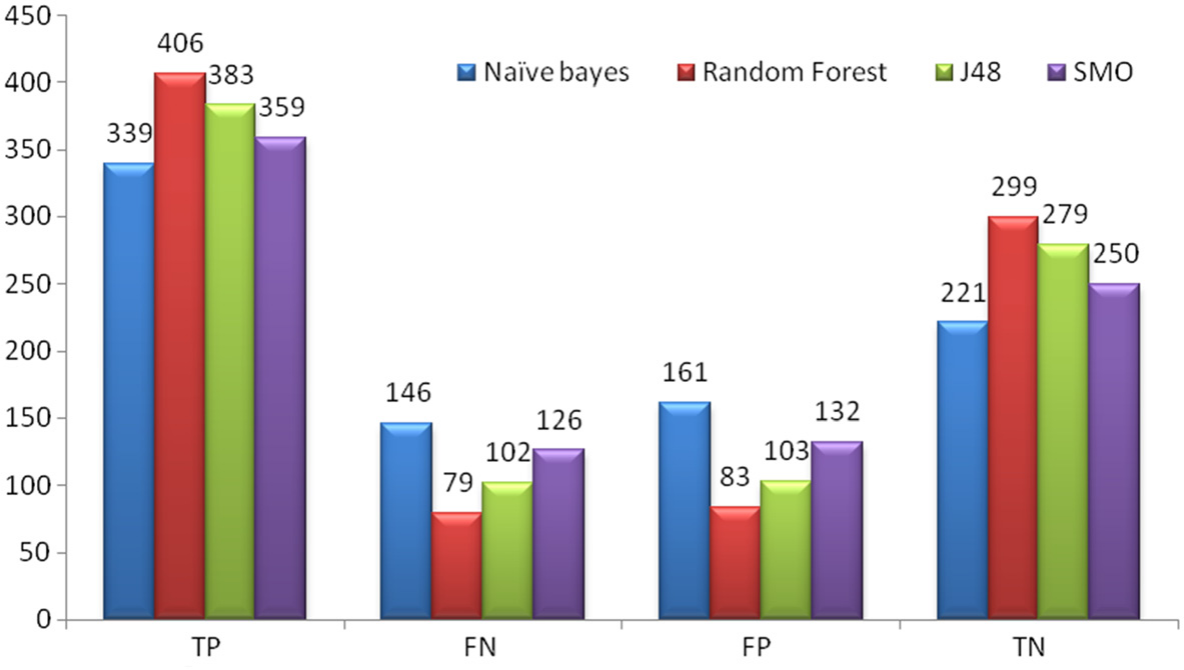
The graph represents number of Set 1 compounds classified by TP, FN, FP and TN by Naive Bayes, Random Forest, J48 and SMO classifiers.

**Set 1:** The results given in Table 2 show that Random Forest outperformed the other classifiers. For Set 1 the Random Forest classifier classified internal 20% test data with 79.81% accuracy, 79.5% average precision and 89.2% AUC ROC which is the best model for the Set 1. For the external set, AID1189, it classified with 64.65% accuracy, 66.4% Average precision and 67.3% AUC ROC as shown in Table 3. For AID1194 it predicted 84.85% accuracy, 84.9% Average precision and 93.1% AUC ROC as shown in Table 4. Figure 2 depicts the number of True Positive (TP), False Positive (FP), True Negative (TN), and False Negative (FN) compounds predicted in Set 1.

**Table 2:** Result table for Set 1 with four classifier algorithms Naïve Bayes, Random Forest, J48 and SMO

***Classifiers***	***TP%***	***FP%***	***TN%***	***FN%***	***Accuracy%***	***Average***	***Average***	***Average***
						***Precision%***	***Recall%***	***ROC***
***Naïve Bayes***	69.9	42.1	57.9	30.1	64.59	67.8	69.89	71.90%
***Random Forest***	**83.7**	**21.7**	**78.3**	**16.3**	**79.81**	**79.5**	**78.3**	**89.2%**
***J48***	79	27	73	21	76.35	78.8	78.96	77.20%
***SMO***	74	34.6	65.4	26	70.24	73.1	74.02	77.10%

Random Forest showed the Best accuracy with 79.81% and ROC Area of 89.2%. It also has high True Positive (TP) rate with low False Positive (FP) rate.

**Table 3:** Result table for AID1189 taken as test set for the models prepared by different sets i.e. Set 1, Set 2 and Set 3

***Classifiers***	***Data set***	***Accuracy%***	***Precision %***	***Recall%***	***ROC***
***Naïve Bayes***	Set 1	49.08	53.3	36.80	50.30%
	Set 2	49.28	53.7	36.29	50.60%
	Set 3	49.01	51.5	49	55.5%
***Random Forest***	Set 1	64.65	66.4	64.7	**67.3%**
	Set 2	61.61	66.6	56.21	**64.50%**
	Set 3	62.89	64	62.9	**65.60%**
***J48***	Set 1	63.16	68.6	57.10	**64.60**%
	Set 2	60.39	66	53.04	62.50%
	Set 3	61.27	62.1	62.3	60.8%
***SMO***	Set 1	50.57	55.3	38.57	55.90%
	Set 2	57.14	63.2	46.95	57.90%
	Set 3	56.12	57	56.1	61.2%

It was found that AID1194 classified better on Set 3 with above 90% accuracy.

**Table 4:** Result table for AID1194 taken as validation set for the models generated on different sets i.e. Set 1, Set 2 and Set 3

***Classifiers***	***Data set***	***Accuracy%***	***Precision %***	***Recall%***	***ROC***
***Naïve Bayes***	Set 1	55.76	54.3	42.78	57.50%
	Set 2	55.88	54.6	42.27	58.00%
	Set 3	61.05	63.2	61.1	66.8%
***Random Forest***	Set 1	84.85	86.3	81	**93.1%**
	Set 2	87.86	87.7	86.58	**94.30%**
	Set 3	90.14	90.1	90.1	**96.8%**
***J48***	Set 1	80.88	79.0	80.50	84.20%
	Set 2	84.37	85.7	80.50	86.20%
	Set 3	87.01	87	87	**88.7%**
***SMO***	Set 1	62.01	62.6	49.62	67.60%
	Set 2	69.23	71.8	57.97	68.70%
	Set 3	56.12	57	56.1	61.2%

It was found that AID1194 classified better on Set 3 with above 90% accuracy.

**Figure 3:**
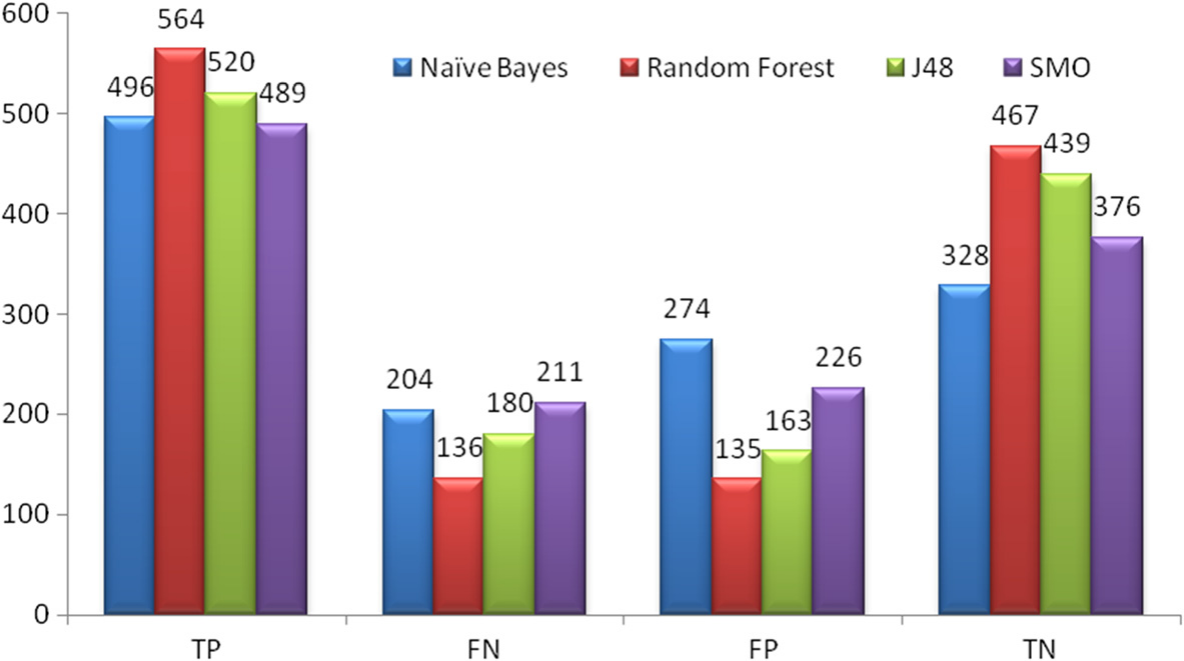
The graph represents number of Set 3 compounds classified by TP, FN, FP and TN by Naive Bayes, Random Forest, J48 and SMO classifiers.

**Set 2:** The results provided in Table 5 portray the predictive power of the Random Forest algorithm. Here it was observed that for Set 2 the Random Forest classified the internal 20% test data with 78.18% accuracy, 77.3% precision and 85% AUC ROC. J48 also performed well with 73.6% accuracy but was not better than Random Forest. With external test set AID1189 it classified with 61.6% accuracy, 66.6% precision and 64.5% ROC area as given in Table 3. With AID1194 it classified 87.86% accuracy, 87.7% precision and 94.3% AUC ROC as given in Table 4. Figure 3 depicts the number of True Positive (TP), False Positive (FP), True Negative (TN), and False Negative (FN) compounds predicted in Set 2.

**Table 5:** Result table for Set 2 with four classifier algorithms Naïve Bayes, Random Forest, J48, and SMO

***Classifiers***	***TP%***	***FP%***	***TN%***	***FN%***	***Accuracy%***	***Precision %***	***Recall%***	***ROC***
***Naïve Bayes***	70.9	45.5	54.5	29.1	63.28	64.4	70.85	69.60%
***Random Forest***	**80.6**	**22.4**	**17.6**	**19.4**	**79.18**	**79.2**	**79.2**	**87.4**%
***J48***	74.3	27.1	72.9	25.7	73.65	74.0	74.28	77%
***SMO***	69.9	37.5	62.5	30.1	66.43	68.4	69.85	78.10%

Random Forest showed the Best accuracy 85.15% with ROC Area 92.4%. It also has high True Positive (TP) rate with low False Positive.

**Figure 4:**
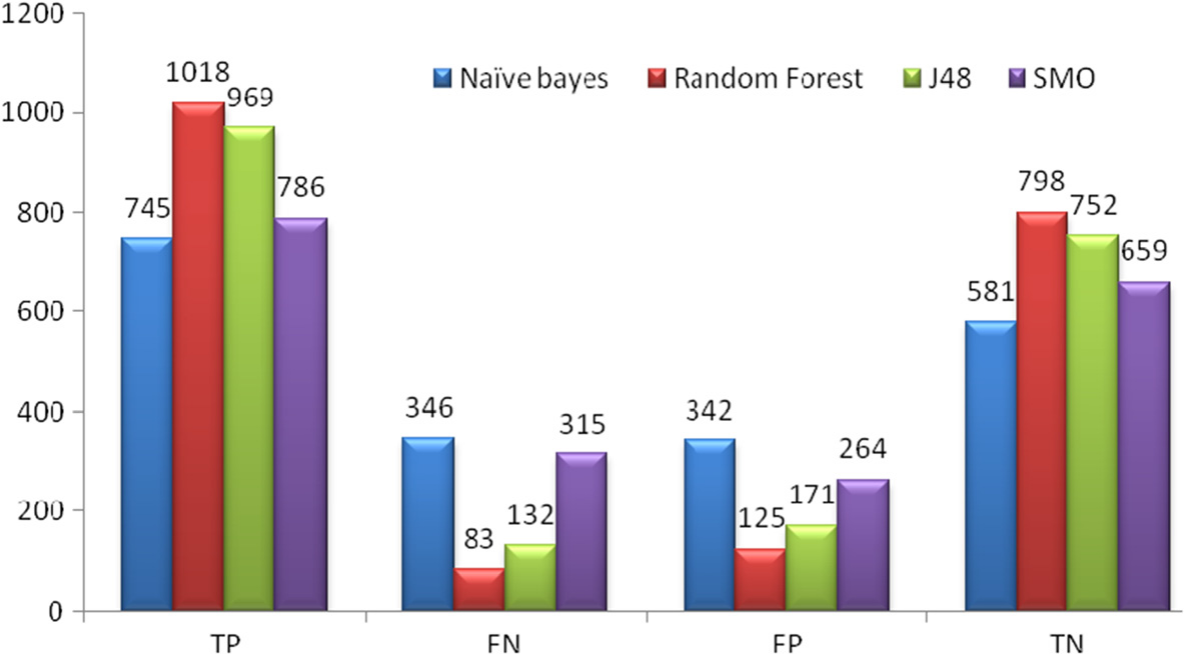
The graph represents number of Set 3 compounds classified by TP, FN, FP and TN by Naive Bayes, Random Forest, J48 and SMO classifiers.

**Set 3:** 154 descriptors were taken from initial 179 descriptors ,which contained 24 272 weighted burden number descriptors, 8 properties 273 descriptors and 124 pharmacophore fingerprints. The results are given in Table 6 classifiers. After merging compounds from the two sets it was observed that Random Forest was better in classifying compounds and for external test sets gave more accurate results than for the other two set (Tables 3 and 4). For the internal 20% test set it gave an accuracy of almost 90% and AUC ROC of 95.3%. For AID1194 it showed an accuracy of 91.9% with ROC area of 96.3%. The other classifier J48 gave an accuracy of 87%. Figure 4 mentions the number of True Positive (TP), False Positive (FP), True Negative (TN), and False Negative (FN) compounds predicted in Set 3.

**Table 6:** Result table for Set 3 with four classifier algorithms Naïve Bayes, Random Forest, J48, and SMO

***Classifiers***	***TP%***	***FP%***	***TN%***	***FN%***	***Accuracy%***	***Precision %***	***Recall%***	***ROC***
***Naïve Bayes***	66.3	28.2	71.8	33.7	68.84	69.3	68.8	75.8%
***Random Forest***	86.7	16.6	83.4	13.3	85.15	85.2	85.2	92.4%
***J48***	83.1	26	74	16.9	78.85	78.9	78.9	80.7%
***SMO***	76.6	34.4	65.6	29.3	71.4	71.5	71.5	78.5%

Random Forest showed the Best accuracy 85.15% with ROC Area 92.4%. It also has high True Positive (TP) rate with low False Positive.

**Figure 5:**
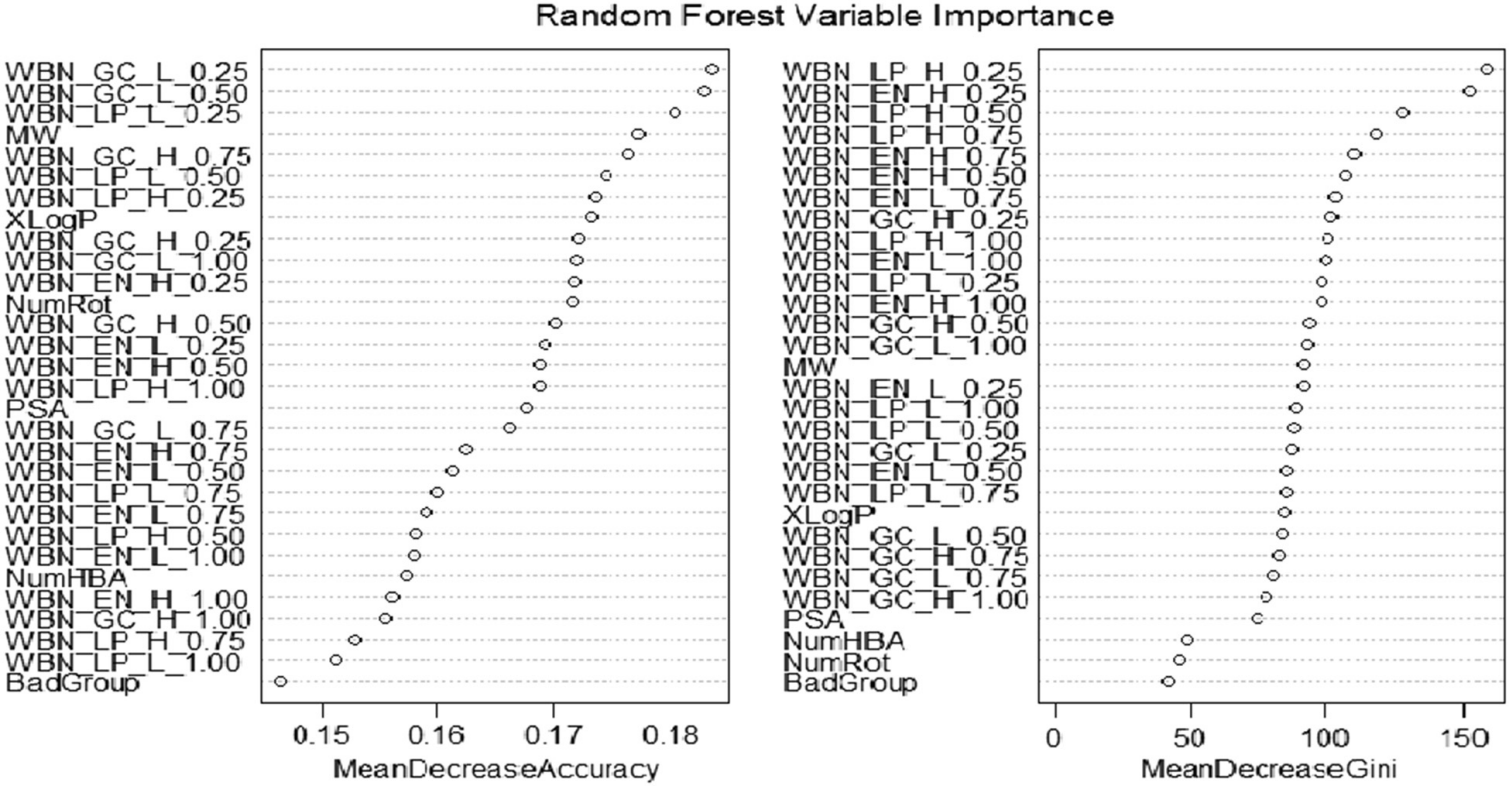
Set 3 Variable Importance Graph.

For each of the data sets modeled with Random Forest performance was much better than the other classifiers. The Random Forest model performs an implicit feature selection, using a small subset of "strong variables" for the classification only, leading to its superior performance on high dimensional data. The outcome of this implicit feature selection of the Random Forest can be visualized by the "Gini importance". In the Figure 5, important variables used in Random forest model generation are represented. We also used the important variables based on Gini Importance i.e. 30 listed in the diagram to model our data sets. For all the test sets of the three sets the accuracy was in range of 79% to 84%. The variable selection using Gini importance resulted in a decrease of accuracy rate to 1% to 1.5%. Descriptor optimization is an important step while making learning models. Descriptors are often selected based on the correlation methodology [32] for example in Weka, a cfs subset Eval attribute evaluator is present which selects the most uncorrelated descriptors for model generation. The Gini importance showed in the Figure 5 is another approach of variable selection which is based on inequality among values of a frequency distribution on each split of the tree [33]. It is defined as a ratio with values between 0 and 1: the numerator is the area between the Lorenz curve of the distribution and the uniform distribution line; the denominator is the area under the uniform distribution line.

We use the models to test the 1410 approved drugs compounds and 66 withdrawn drugs and also 22080 metabolites in the ZINC database. It was assumed that the approved drug compounds would not show mutagenicity and hence, were labeled as non-mutagens. The withdrawn compounds show various pharmacological side effects and among them mutagenicity could also be an important side effect. So, the withdrawn compounds were labeled as mutagens. Among the metabolites 9523 compounds were labeled as mutagens and remaining as non-mutagens arbitrarily. We tested the compounds on the three sets with Random Forest of 100 trees. The Table 7 displays the tested compounds results.

**Table 7:** The drug and the metabolites data tested with Set 1, Set 2, Set 3 with random forest

***Datasets***	***True Positives***	***False Negatives***	***True Negative***	***False Positives***
Set1 (Drug Data)	16.7	83.3	84.6	15.4
Set1(Metabolites)	17.2	82.8	84	16
Set1(Cost sensitive classification of Drug data)	13.6	86.4	90.2	9.8
Set2 (Drug Data)	19.7	80.3	84.4	15.6
Set2 (Metabolites)	16.6	83.4	85.3	14.7
Set2 (Cost sensitive classification of Drug data)	12.3	87.7	91	9
Set3(Drug Data)	21.2	78.8	85.3	14.7
Set3 (Metabolites)	15.6	84.4	85.8	14.2
Set3 (Cost sensitive classification of Drug data)	12.1	87.9	90.8	9.2

Each model was tested with the drug data and the metabolites data. It was found that every model predicted the drug data with almost the same specificity i.e. the true negatives which were labeled as non- mutagen. Every model predicted with almost more than 84% specificity. To improve the model of prediction of true negatives we also implemented the classification with cost matrix in Weka and tested our data sets. We set the cost of false positive to 2.5 for misclassifying every non-mutagenic compound. Every data set was classified with more than 90% as true negative. The models predicted the withdrawn drugs data with low sensitivity and it predicted most of the compounds as false positives (non-mutagen). The compounds from Zinc metabolites database show very low mutagenic effects to the living systems and after testing with each model it was observed that Set 3 gave the best classification of the compounds. From 9523 mutagen compounds labeled arbitrarily, it predicted 8037 compounds as false negatives (mutagens compounds labeled predicted as non- mutagens) and 10774 compounds as True negatives (non-mutagens compounds labeled predicted as non-mutagens) from 12557 compounds. This indicates that 85% of the compounds in the zinc metabolite dataset are non-mutagenic.

### Analysis of false positives and false negatives results

Erroneous compounds i.e. the false positives, false negatives were observed for the test set of Set 3, drug data sets, and metabolites. Each data set is described below.

**Figure 6:**
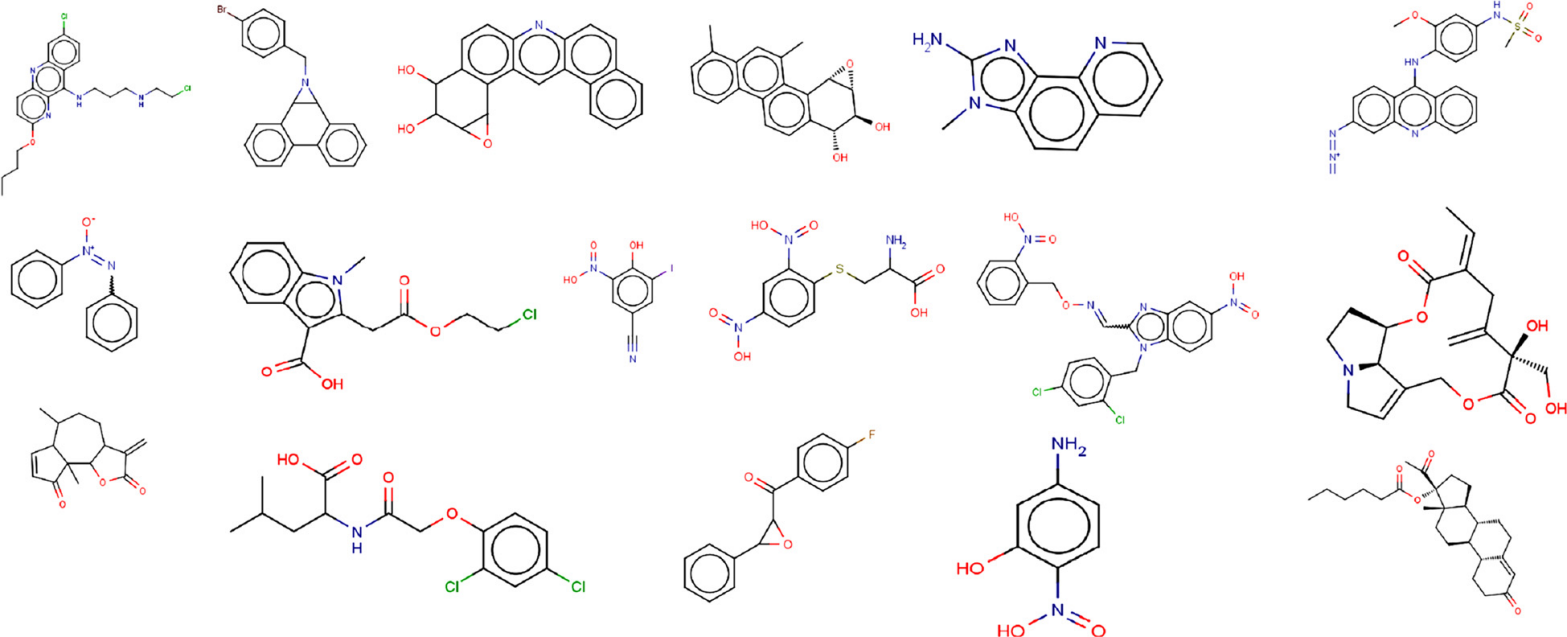
Represents some compounds which are Mutagenic but predicted as Non Mutagen(False Negative) by Random Forest in the test set.

**Figure 7:**
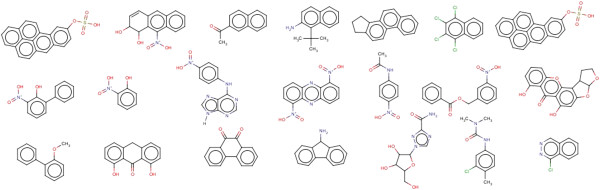
Shows false positive compounds of the test sets.

Set 3: The test set contained 1698 compounds of which 910 compounds were classified as mutagens and 788 as non-mutagens. False Negatives (Mutagenic compounds incorrectly classified as Non-Mutagens) appeared in the test sets which resulted in 121 compounds. It was observed that compounds containing toxicophores were being classified as non-mutagens. From 121 false negatives, derivatives of 18 aromatic nitro groups, 9 quinoline, 7 butyl acetate, 5 cresol, 4 phenanthrene, 4 acetanilide, 3 carbinol, 3 methyl aminoethanol, 3 azo compounds were observed and the remaining were singletons. Some of the compounds are given in Figure 6. 131 false positives (non mutagenic compounds incorrectly classified as mutagenic) compounds were also predicted by the Random Forest classifier. It was observed that 14 aromatic nitro groups, 21 styrene groups, 4 anisoles, 4 benzylamines, 4 dimethylaniline, containing compounds were predicted as mutagenic due to presence of aromatic nitro group, 3 quinolines. Additional file 4 contains the smiles and the predicted results of false positives and false negatives of the test set. Figure 7 shows some false positive compounds.

**Figure 8:**
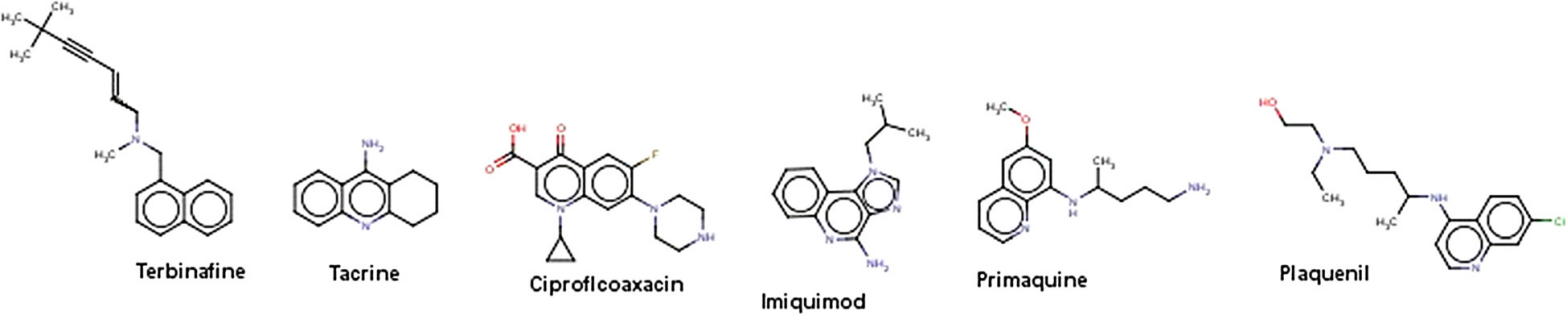
Shows some drugs predicted as false positives.

DrugBank data set: It was observed that 207 compounds where predicted as mutagens among 1410 approved drugs. It was found that compounds containing some essential toxicophores were classified as mutagenic. The structures present in the Figure 8 are drugs which are predicted as false positives. For example, Tacrine which was used to treat Alzheimer’s disease is a centrally active acetyl cholinesterase inhibitor but it was also observed that it induces reversible increase in transaminase activity leading to hepatic injury to 30-50% of the patients [34]. Ciprofloxacin a flourinated quinoline belonging to the class of antibiotics which includes other drugs such as the enoxacin, fleroxacin, norfloxacin, ofloxacin etc. Ciprofloxacin is associated with fatal liver failure [35] and also it has been associated with cases of renal failure. This drug has been predicted as false positive which indicates that the model predicted the compound which was labeled non mutagen is originally mutagenic.

**Figure 9:**
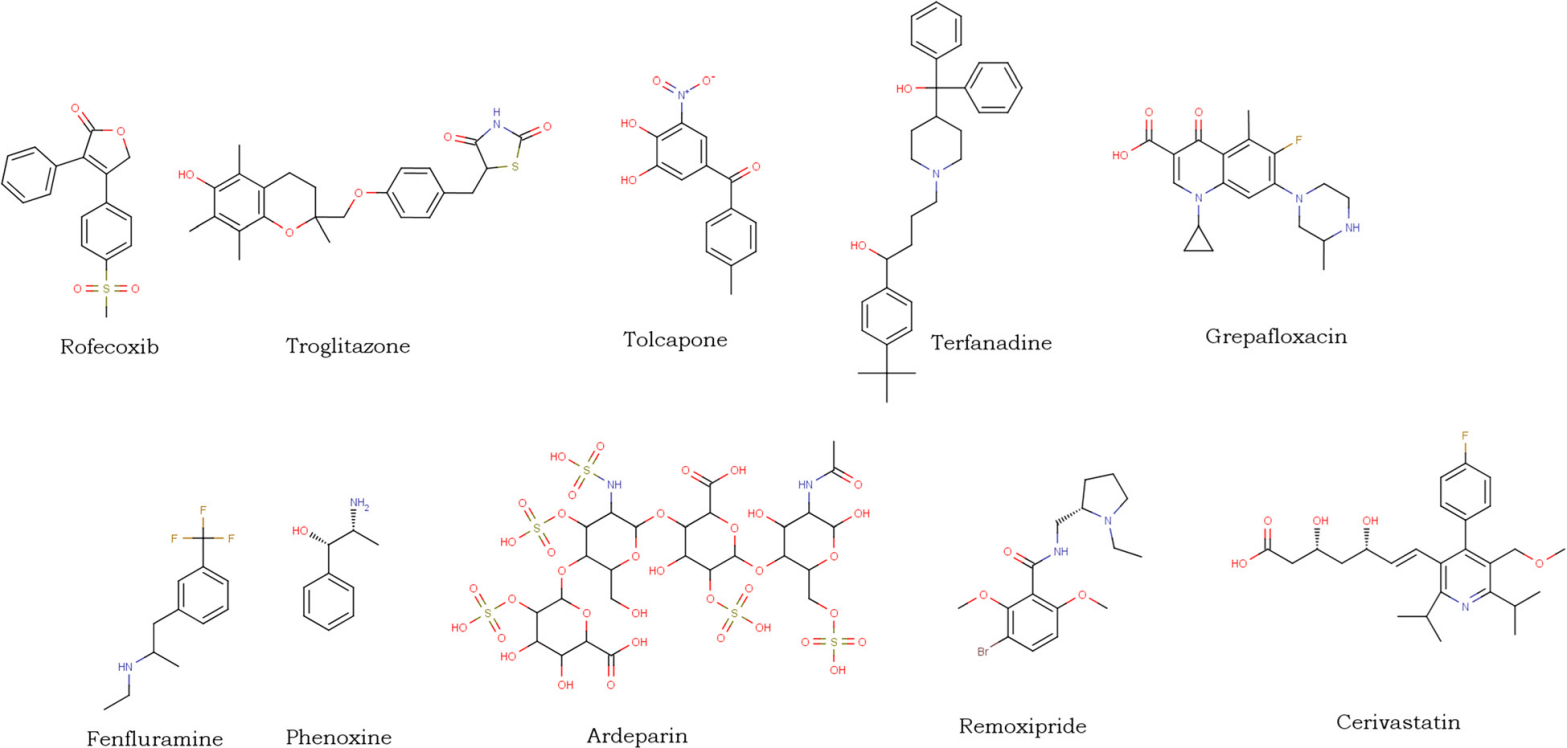
Shows the withdrawn drug compounds predicted as false negative.

Of the 66 withdrawn drugs labeled as mutagens, only 14 compounds were identified as mutagens. The drugs were withdrawn from the market due to signs of toxicity and adverse effects to humans. Side effects include hepatotoxicity, hepatitis, teratogenicity (study of human birth defects), myocardial infarction, mutagenicity and others. In the withdrawn data 52 compounds were predicted by Random Forest as non mutagens. Figure 9 shows some of the compounds which are predicted as false negatives. Additional file 5 contains smiles and the predicted results of approved and withdrawn compounds.

Metabolites data set: This data set contained 22080 compounds and around 3269 compounds were predicted as mutagens. The Additional file 6 contains the ZINC ids and smiles along with predictions of the Random Forest Set 3 classifier.

### Comparison of the random forest with CAESAR

The results of the Random Forest classifier were compared with the standalone CAESAR mutagenicity software (v.2.0). The results are provided in the Table 8. It was observed that CAESAR was unable to predict certain compounds that contained ions in their structure. For the validation sets AID1194, AID 1189 and the test sets of Set 3, the total number of predicted mutagens were 394,788 and 910 respectively. The non-mutagens predicted to be were 438,697 and 788 for the above sets. The CAESAR tool is based on the structural alerts described by Ashby, Kazius et al. [36]. The tool was unable to predict correctly 163,322,124 compounds for AID 1194, AID1189 and 20% of test set respectively in the validated datasets. The results in the table show number of classified compounds with respect to the total number of compounds in each of the data sets. The comparison clearly shows that our Random Forest model performed much better than CAESAR and could even classify compounds which are not classified by the tool.

**Table 8:** Comparison of Caesar with Random Forest (rf) with the validation sets depicting True Positives (TP), False Negatives (FN), True Negatives (TN), False Positives (FP) and Accuracy

	**TP**	**FN**	**TN**	**FP**	**Accuracy**
AID 1194(caesar)	277/394	44/394	289/438	59/438	68.02
AID 1194(rf)	350/395	45/395	400/437	37/437	90.1
AID1189(caesar)	399/788	266/788	334/697	164/697	49.3
AID1189(rf)	436/788	352/788	493/697	204/697	62.9
20%test(caesar)	752/910	113/910	558/788	151/788	77.1
20%test(rf)	789/910	121/910	657/788	131/788	85.15

## Conclusion

Previously the Benchmark data set was the largest mutagenicity data set containing more than 6000 molecules classified as mutagens and non-mutagens. In this work we were able to create a new mutagenicity data set (Set 3) containing more than 8000 compounds.

The models generated using Random Forest classifier was observed to have a high performance rate. This was proved by a higher sensitivity and specificity results for the validation sets AID1189, AID 1194. Descriptor optimization is important criteria for model generation, the use of Gini importance could play an important role in descriptor space optimization. Other than that the comparative results of descriptor based Random Forest with CAESAR (which is based on the structural alerts) clearly shows that Random Forest has the better predictive ability to classify mutagenic from non-mutagenic. Classification of the Drug data and the metabolite datasets gave us a clear view the impact of predictive models in drug design and discovery. The mutagenic predictive models could make a great impact in classifying compounds in large repositories such as PubChem and ZINC which could help to accelerate the pipeline of drug discovery.

## Competing interests

The authors have no competing interests in this paper.

## Authors’ contributions

We would like to mention that Mr. AS and Mr. AP contributed equally to this work and are the first authors of this paper with Mr. AS being the corresponding author s well. Dr. UCAJ, Dr. DJW, OSDD Consortium are the co-authors of this paper. Dr DJW & Dr UCAJ helped in editing the manuscript. All authors read and approved the final manuscript.

## Supplementary Material

Additional file 1 The Mutagenic training set. Click here for file

Additional file 2 The Mutagenic test set. Click here for file

Additional file 3 CSV format file of descriptors for External dataset drugs and metabolites. Click here for file

Additional file 4 False positive and negative compounds of the test set. Click here for file

Additional file 5 False positive and false negative compounds of the DrugBank approved and withdrawn drugs. Click here for file

Additional file 6 Predicted metabolites from ZINC dataset. Click here for file
